# Effect of laser corticotomy on canine retraction rate: a split-mouth randomized clinical trial

**DOI:** 10.1186/s12903-024-04192-y

**Published:** 2024-04-12

**Authors:** Mohammad Hossein Toodehzaeim, Fahimeh Rashidi Maybodi, Elaheh Rafiei, Pedram Toodehzaeim, Negin Karimi

**Affiliations:** 1 Department of Orthodontics, School of Dentistry, Shahid Sadoughi University of Medical Sciences, Yazd, 8916978477 Iran; 2 Department of Periodontics, School of Dentistry, Shahid Sadoughi University of Medical Sciences, Yazd, 8916978477 Iran; 3 Department of Orthodontic, University of British Columbia, Vancouver, Canada; 4 Department of Orthodontic, School of Dentistry, Kerman University of Medical Science, Kerman, 7616913555 Iran

**Keywords:** Tooth movement, Lasers, Maxilla, Bicuspid, Orthodontics

## Abstract

**Background:**

This study assessed the effect of corticotomy with Er: YAG (erbium-doped yttrium aluminium garnet) laser on the rate of canine retraction.

**Methods:**

This randomized split-mouth controlled clinical trial was conducted on 12 patients undergoing orthodontic treatment with extraction of maxillary first premolars. Following initial leveling and alignment, an alginate impression was made from the maxillary arch, and Er: YAG laser corticotomy was performed in one of the maxillary quadrants of each patient. Canine retraction was started immediately after corticotomy by placement of nickel-titanium (NiTi) closed coil springs at both sides. At the end of each month, alginate records were repeated for 4 months. Study models were scanned, and the anteroposterior movement of canine was quantified bilaterally. Pain was also measured by a visual analog scale (VAS). Probing depth (PPD) of canines and two adjacent teeth was also evaluated and pulp vitality was assessed by performing the cold test. Data were analyzed by paired and independent t-test and one-way ANOVA (alpha = 0.05).

**Results:**

The rate of canine retraction was significantly greater in the laser-assisted corticotomy quadrant than the control (*P* < 0.05). No significant difference existed in posterior anchorage loss, canine rotation angle, PPD, pulp vitality, or pain score between two groups (*P* > 0.05).

**Conclusions:**

Flapless Er: YAG laser corticotomy significantly enhanced canine retraction rate with no adverse effect on other parameters.

## Introduction

Fixed orthodontic treatment often takes 2 to 3 years; such a long course of treatment increases the risk of enamel demineralization, external root resorption, and gingival inflammation, and decreases the cooperation of patients, which can compromise the treatment outcome [1–3]. Also, the majority of adult patients wish to accomplish their orthodontic treatment in the shortest possible time due to social and esthetic concerns [4]. Therefore, several strategies are currently adopted by orthodontists to expedite the speed of orthodontic tooth movement (OTM) and improve the treatment efficacy with no adverse effect on the outcome [5].

In an attempt to enhance OTM, several studies evaluated the biological mechanism of OTM and the possibility of its physiological manipulation. OTM is an inflammatory process.

Induction of inflammation to accelerate OTM is not a novel idea. Kole in 1959 suggested to connect corticotomies of the vestibule and lingual segment and perform sub-apical osteotomy after a complete periosteal flap elevation. Kole further expanded the bone block theory to explain faster OTM. In the next years, some simplified protocols without subapical osteotomy were developed; however, they still highly depended on the bone block theory [6].

Frost was the first to use the term regional acceleratory phenomenon (RAP) to describe tissue reaction to a harmful stimulus through production of inflammatory mediators in 1983 [7].

Many attempts have been made to locally accelerate OTM, and several surgical and non-surgical interventions have been attempted to induce inflammation and accelerate OTM. Low-level lasers, pulsating electromagnetic fields, corticotomy and distraction osteogenesis are among the attempted approaches [8–14].

Several corticotomy methods have been previously used to shorten the course of orthodontic treatment [11, 15]. The accelerating effect of corticotomy in the first place is related to the RAP [12, 16]. Moreover, corticotomy can induce the expression of pro-inflammatory markers and cytokines that activate the activity of osteoclasts [15].

Although the corticotomy techniques can induce the initiation of OTM [15], they are often relatively invasive since they require elevation of a complete mucoperiosteal flap and suturing, and are associated with pain [17], edema, and slight reduction of interproximal bone and the attached gingiva [17]. Thus, they did not gain popularity among orthodontists [15]. Therefore, in the recent years, less invasive and more conservative flapless corticotomy techniques were introduced such as corticision, piezocision, micro-osteoperforation [18, 19], and flapless laser corticotomy [20, 21]. It has been claimed that the speed of OTM increases following minimally invasive surgical procedures. Also, such procedures have insignificant side effects. However, studies in this respect are scarce, and clinical trials are required to better elucidate this topic [15]. Thus, this study aimed to assess the effect of corticotomy with erbium-doped yttrium aluminum garnet (Er: YAG) laser on the rate of canine retraction. The null hypothesis was that the rate of canine retraction, amount of anchorage loss and amount of canine rotation during retraction are not affected by laser corticotomy.

## Methods

This study was conducted at the Orthodontics Department of School of Dentistry, Yazd Shahid Sadoughi University of Medical Sciences in 2021. The study protocol was approved by the ethics committee of the university (IR.SSU.REC.1400.028), and registered in the Iranian Registry of Clinical Trials (IRCT20210531051460N1- Registered at 27/07/2021).

### Trial design

A split-mouth randomized controlled clinical trial was designed in which the experimental side underwent laser corticotomy before canine retraction while canine retraction was performed without laser corticotomy in the control side. The results were reported in accordance with the Consolidated Standards of Reporting Trials (CONSORT).

### Participants, eligibility criteria, and settings

The inclusion criteria were orthodontic patients between 15 and 30 years with Class I malocclusion who required orthodontic treatment with bilateral extraction of maxillary first premolars due to severe crowding or dental protrusion or patients with Class II malocclusion with dentoalveolar maxillary protrusion requiring bilateral extraction of maxillary first premolars (camouflage treatment), presence of vital teeth with normal periodontium and no root resorption, presence of both maxillary first and second premolars, no systemic diseases such as severe renal or hepatic disease, immunocompromising conditions, hematological diseases, diabetes mellitus, vitamin D deficiency, hyperparathyroidism, and osteoporosis. No syndromes or craniofacial deformity, no intake of medications affecting OTM during orthodontic treatment, no history of previous orthodontic treatment, normal canine tooth morphology and O’Leary plaque Index < 30%. The exclusion criteria were bracket debonding during canine retraction, discontinuation of treatment for any reason, not showing up regularly for continuation of treatment.

According to a previous study [21], the minimum sample size was calculated to be 12 in each group, who were selected among those presenting to the Orthodontics Department by convenient sampling.

### Informed consent

Informed consent was obtained from the patients to participate in the study and to use the results obtained from the study by the post-graduate student.

### Interventions

#### Orthodontic treatment prior to corticotomy

After obtaining written informed consent from all patients, they received oral hygiene instructions prior to orthodontic treatment, and underwent extraction of maxillary first premolars prior to banding and bonding. Banding and bonding were then performed, and maxillary second molars were also banded. The leveling and alignment phase was started as such. Extraction of maxillary first premolars at the onset of treatment was performed to accelerate leveling and alignment of teeth and eliminate the effect of RAP following tooth extraction, and subsequent assessment of the pure effect of laser corticotomy on canine tooth movement. The MBT system with 22 × 28-inch slot size was used for orthodontic treatment of patients. Canine retraction was not started until completion of leveling and alignment and reaching 0.019 × 0.025-inch stainless steel wire, which is the working wire for space closure.

#### Laser corticotomy

In the session where the patient was scheduled for corticotomy, before the intervention started (T0), the O’Leary Plaque index was reported by calculating the ratio of tooth surfaces stained with disclosing agent to the total tooth surfaces and then converting it into a percentage. The highest acceptable plaque index for study inclusion was 30% at the onset of canine retraction. The pocket probing depth (PPD) and pulp vitality status of maxillary right and left lateral incisors, canines, and second premolars were also assessed. Next, an alginate impression was made from the maxillary arch as the baseline record.

After completion of leveling and alignment and reaching 0.019 × 0.025-inch stainless steel wire, and prior to canine retraction, laser corticotomy was performed in one randomly selected maxillary quadrant in a split-mouth design. Local anesthesia was administered by injection of lidocaine and 1:80,000 epinephrine at the corticotomy site. The incision depth and gingival thickness at the respective site were measured by a Williams probe such that the soft tissue and 2–3 mm of cortical bone were incised. Laser corticotomy was then performed in the attached gingiva using Er: YAG laser (LightWalker®ST-E, Fotona, Ljubljana, Slovenia) with 2940 nm wavelength and 2 W power with 100 mJ energy and 10 Hz frequency under air and water spray in medium-short pulse (MSP) mode. Next, the laser settings were changed for cortical bone perforation, such that 3 W power, 200 mJ energy, and 12 Hz frequency with air and water spray and quantum-square pulse (QSP) mode were used. The surgical procedure was performed in the buccal at equal distance from the canine and second premolar teeth using the fiber tip of the device. Three perforations were made at the region. The penetration depth was continuously monitored by a Williams periodontal probe.

#### Treatment phase after corticotomy

**Fig. 1:**
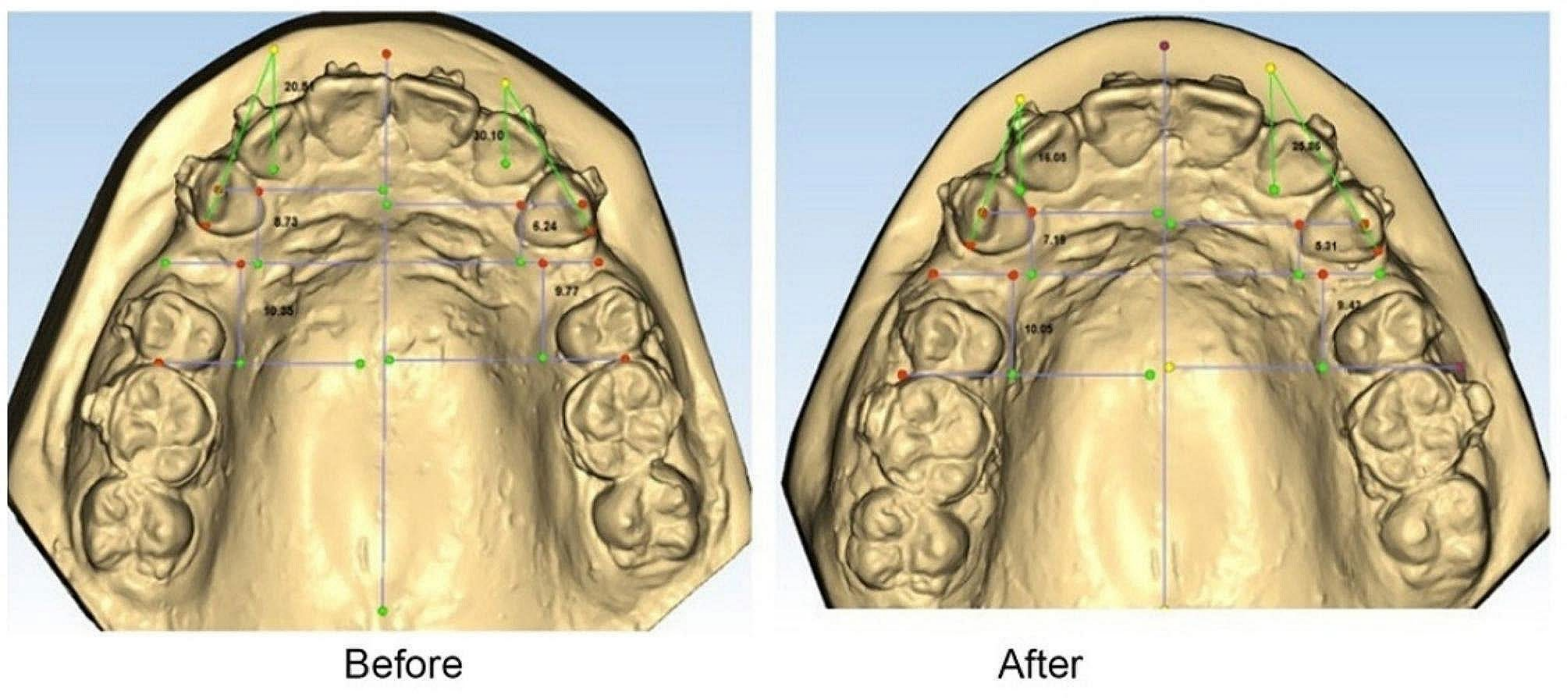
Palatal ruga and mid-palatal raphe lines were identified as the reference lines. Lines locating the canine and molar teeth were also drawn. Distance between these lines and reference lines was measured before and after the intervention, and the difference indicated the magnitude of OTM

To benefit from the RAP to the maximum level, force application to canine tooth was performed every 14 days, instead of every 4–6 weeks. Austenitic NiTi coil springs (G&H Wire Company) with 150 g force, as measured by a force gauge, were used from the bracket hook of canine tooth to the hook of first molar band for canine retraction. To benefit from the RAP (that occurs following bone injury), canine retraction was started immediately after surgery. Due to the bactericidal effects of laser, antibiotic therapy was not required. The patients were provided with the Faces visual analog scale (VAS) and were asked to report their level of pain in the first week after corticotomy. One month after the onset of canine retraction, the arch-wire was removed, and another alginate impression was made. Subsequently, 150 g load was applied again for canine retraction. At the end of the first month, in addition to making an alginate impression, PPD was measured again, and the vitality tests of lateral incisor, canine, and second premolar teeth were repeated. At the end of the second, third, and fourth months following canine retraction, alginate impressions were made again. Study models poured before and after canine retraction were then scanned by a 3D scanner (Maestro3D, MDS500 Dental Scanner). On the scan, the reference lines included the mid-palatal raphe (midline) and the palatal ruga line drawn from the midpoint of the right third palatal ruga. Evidence shows that measurement of OTM by using the third palatal ruga is as reliable as the measurements made by cephalometric superimposition [22]. To measure the anteroposterior canine movement, a line was drawn from the midpoint of the right third palatal ruga perpendicular to the midline (mid-palatal raphe). Also, another line was drawn from the canine cusp tip perpendicular to the midline. The distance between these two lines was measured. Also, lines were drawn from the mesial contact point of the permanent first molar and the palatal ruga line perpendicular to the midline, and the distance between these two lines was measured to assess the movement of molar tooth and posterior anchorage loss. The angle formed between the midpalatal raphe and a line passing through the mesial and distal borders of the canine tooth was also measured to quantify the magnitude of canine rotation (Fig. 1).

### Outcomes (primary and secondary)

The main objective of this study was to assess the effect of corticotomy with Er: YAG laser on the magnitude and speed of canine retraction. Posterior anchorage loss, PPD, pain score, and vitality of lateral incisor, canine and second premolar teeth were also assessed as the secondary outcomes.

### Sample size calculation

The minimum sample size was calculated to be 12 in each group assuming 95% confidence interval, 80% study power, standard deviation of the magnitude of OTM to be 0.4 mm, and a difference of 0.7 mm in the mean OTM between the control and laser groups using the sample size calculation formula.

### Interim analyses and stopping guidelines

No interim analyses were performed, and no stopping guidelines were established.

### Randomization

A number was assigned to all the people who have had their first premolars extracted and were in the pre-retraction stage. Then we chose the starting point in the randomization table with our eyes closed and moved on the same row or column and the numbers that were smaller than the total number of people, were chosen to reach the desired sample size of 12. Sealed envelopes were used to randomize which side of was performed with laser application.

### Blinding

In this study, the person evaluating the amount of canine retraction and the person analyzing the data were not aware of which side the laser intervention was performed and which side was the control. In this study, due to the nature of the intervention, we faced the limitation of blinding the participants.

### Statistical analysis

Data were analyzed using SPSS version 25. The Kolmogorov-Smirnov test was applied to analyze the normality of data distribution. Accordingly, paired t-test, independent t-test, and one-way repeated measures ANOVA were applied for statistical analysis of the data. Due to non-normal distribution of the PPD data, comparisons in this regard were made using the Mann-Whitney test. The Chi-square test was applied to analyze the pain score and pulp vitality. *P* < 0.05 was considered statistically significant.

### Ethical considerations

This study was conducted at the Orthodontics Department of School of Dentistry, Yazd Shahid Sadoughi University of Medical Sciences in 2021. The study protocol was approved by the ethics committee of the university (IR.SSU.REC.1400.028), and registered in the Iranian Registry of Clinical Trials (IRCT20210531051460N1-27/07/2021).

After explaining treatment protocol to patients twelve patients were enrolled after obtaining their informed consent.

## Results

### Participant flow

**Fig. 2:**
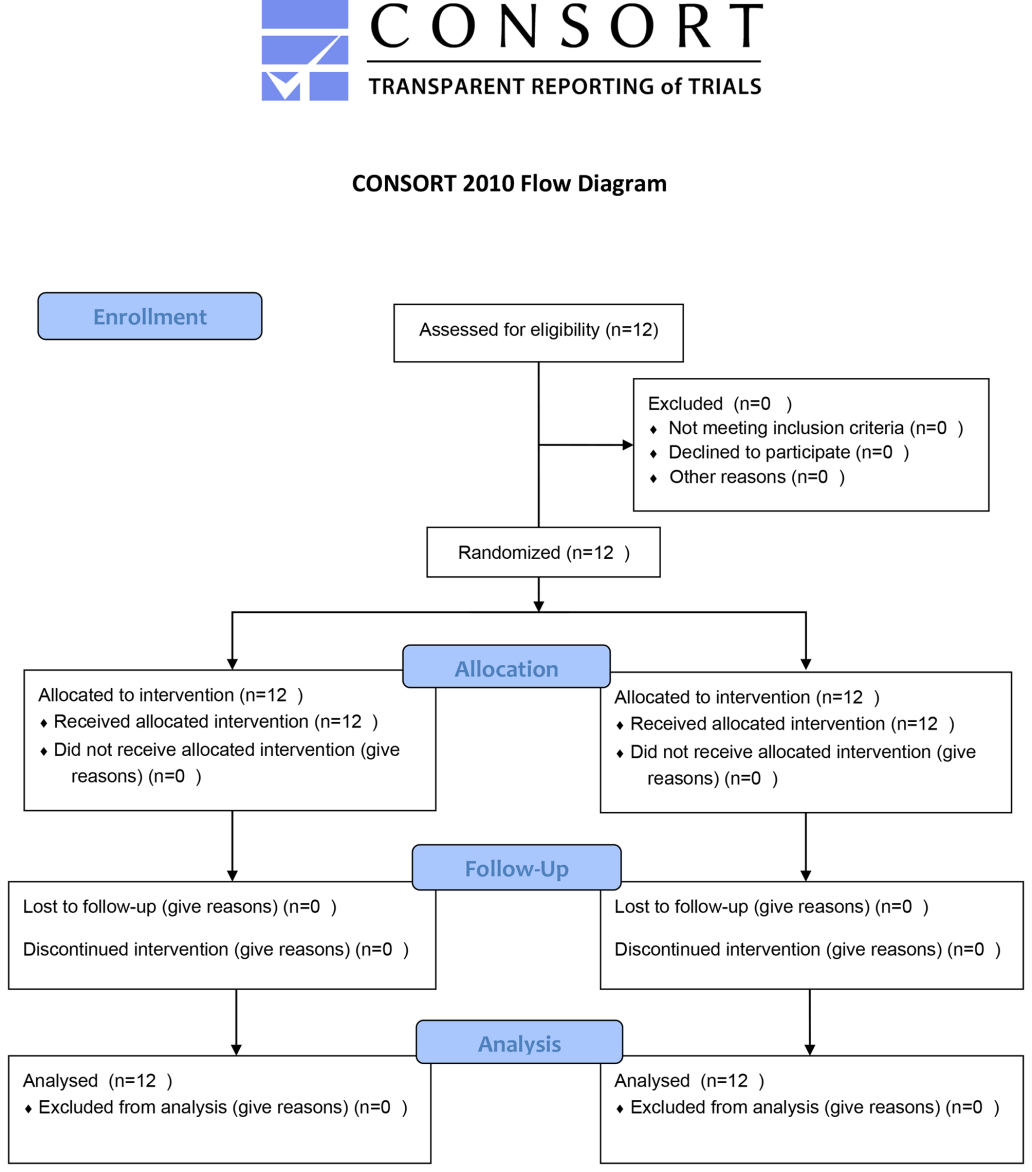
CONSORT flow-diagram of patient selection and allocation

The sample consisted of 7 females and 5 males between 15 and 30 years. The patients were followed up for 4 months. Figure 2 shows the CONSORT flow-diagram of patient selection and allocation. The experimental and control groups had no significant difference in any parameter at baseline (*P* > 0.05).

### Harms

No patients were harmed during the study.

### Subgroup analyses

#### Primary outcome

##### Magnitude of canine retraction

**Fig. 3:**
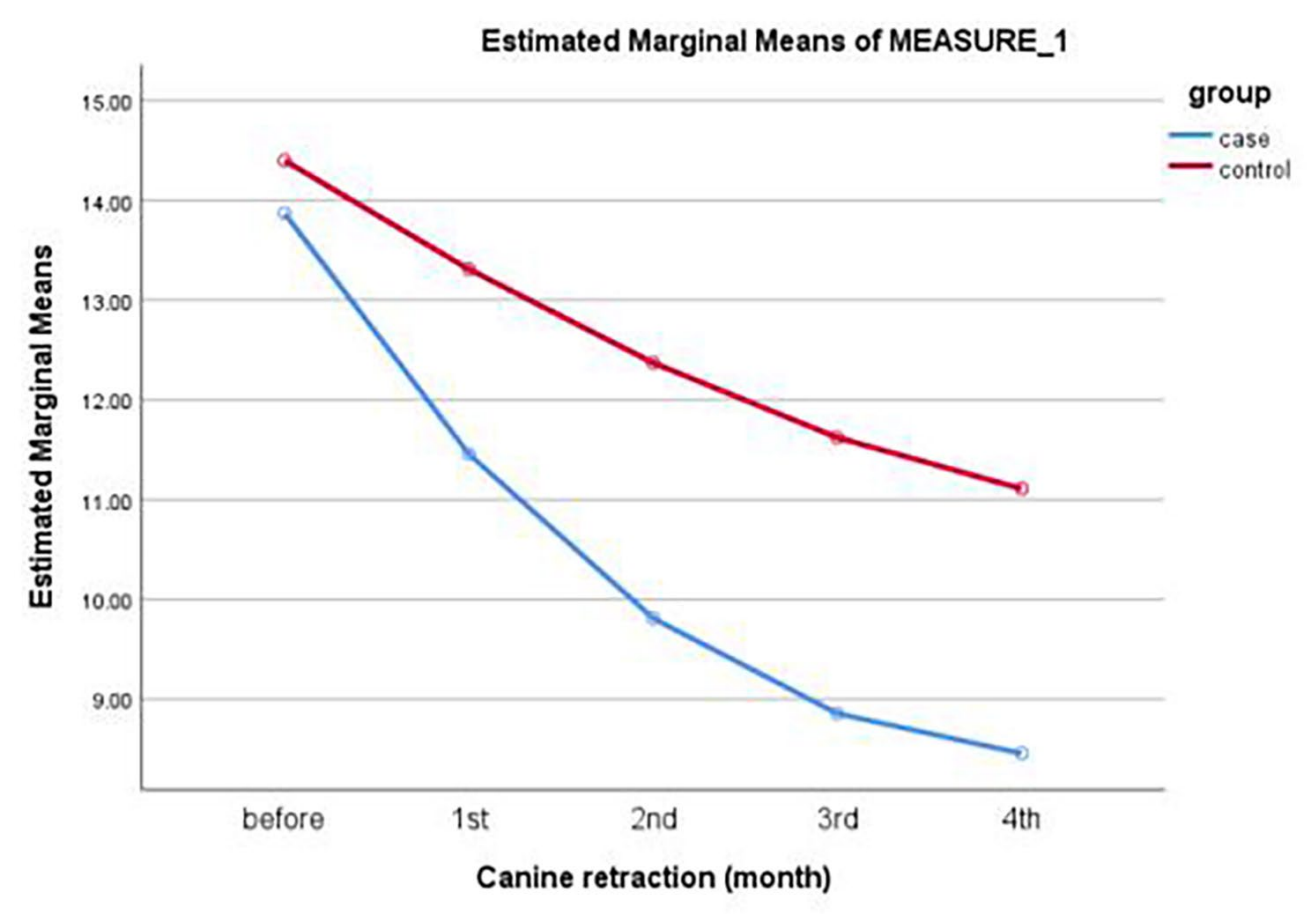
Magnitude of canine retraction (mm) over time in the two groups

Table 1 shows the magnitude of canine retraction over time in the two groups. One-way repeated measures ANOVA showed that the magnitude of canine retraction was significant over time in both groups (*P* = < 0.05). Also, paired t-test showed a significant difference in the magnitude of canine retraction between the two groups at all-time points. As the distance between the line from the midpoint of the right third palatal ruga perpendicular to the midline (midpalatal raphe) and the line from the canine cusp tip perpendicular to the midline was significantly lower in corticotomy group at all-times, so it shows that the magnitude of canine retraction was significantly greater in the corticotomy group (*P* < 0.05) (Fig. 3).

**Table 1:** Magnitude of canine retraction (mm) over time in the two groups

Group	Time 0 Mean ± SD	Time 1 Mean ± SD	Time 2 Mean ± SD	Time 3 Mean ± SD	Time 4 Mean ± SD
Corticotomy group	± 0.82 13.87	± 0.91 11.45	± 1.06 9.81	± 1.31 8.85	± 1.39 8.45
Control group	± 0.96 14.39	± 1.10 13.31	± 1.30 12.37	± 1.40 11.62	± 1.54 11.10
*P*-value	0.31	0.009	< 0.05	0.001	0.003

One way repeated measures ANOVA

Time 0: Before laser corticotomy; Time 1: 1 month after laser corticotomy, Time 2: 2 months after laser corticotomy, T3: 3 months after laser corticotomy, Time 4: 4 months after laser corticotomy

##### Speed of canine retraction

As shown in Table 2, independent t-test revealed that the speed of canine retraction (mm/month) was significantly higher in the corticotomy group than the control group.

**Table 2:** Speed of canine retraction in the two groups

Time	Corticotomy group Mean ± SD	Control group Mean ± SD	Mean Diff. (95%CI)	*P* value
T0-T1 (First month)	± 0.30–2.36	± 0.64–1.05	-1.31	< 0.05
T1-T2 (Second month)	± 0.41–1.58	± 0.40–0.80	-0.78	< 0.05
T2-T3 (Third month)	± 0.37–0.81	± 0.27–0.66	-0.14	0.29
T3-T4 (Fourth month)	± 0.15–0.40	± 0.24–0.51	0.11	0.29

Independent t-test

Time 0: Before laser corticotomy; Time 1: 1 month after laser corticotomy, Time 2: 2 months after laser corticotomy, T3: 3 months after laser corticotomy, Time 4: 4 months after laser corticotomy

#### Secondary outcomes

##### Posterior anchorage loss

**Fig. 4:**
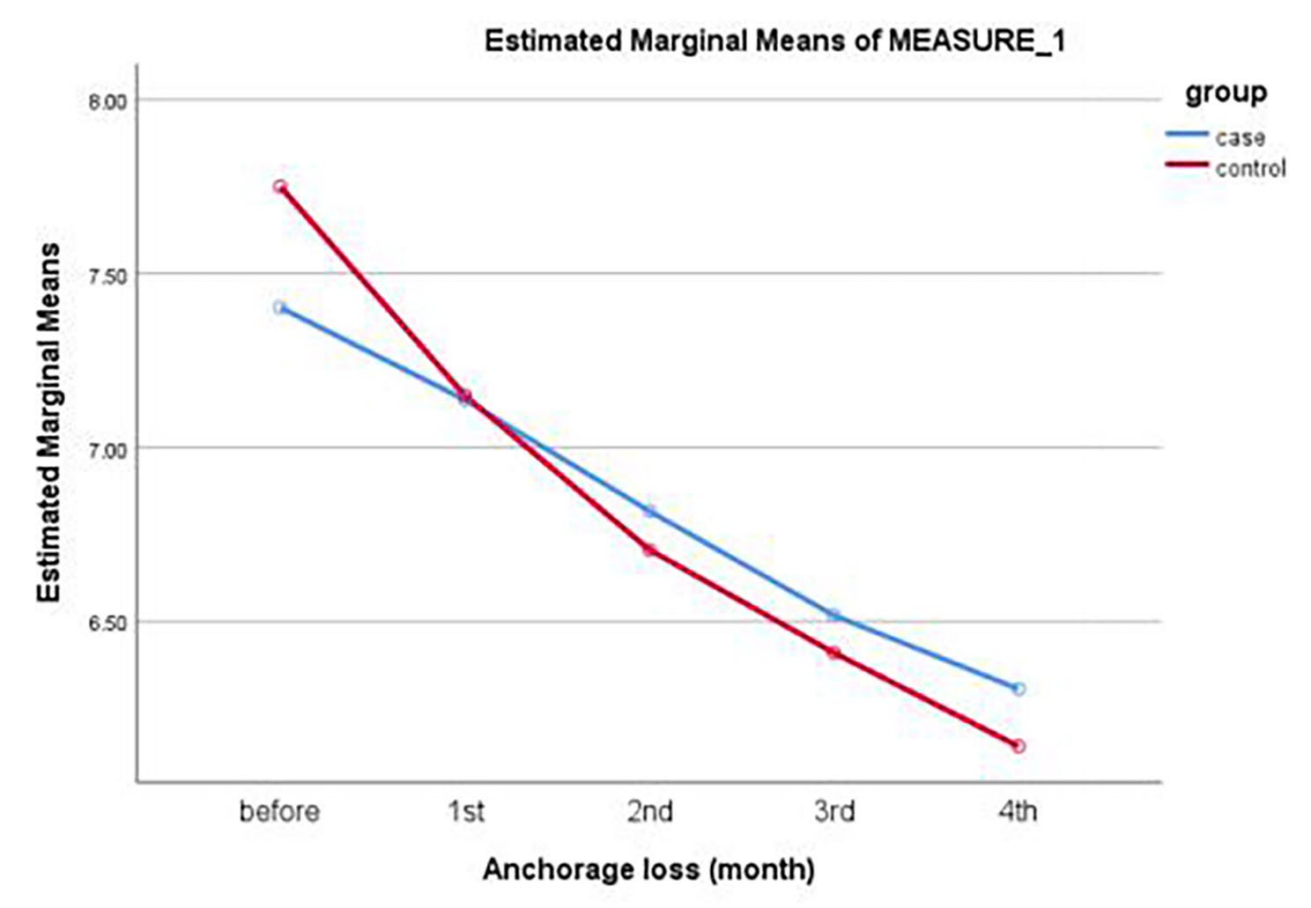
Posterior mean anchorage loss in the two groups at different time points

One-way repeated measures ANOVA showed that the change in posterior anchorage loss was not significant in any group (*P* = 0.21). Paired t-test showed no significant difference between the two groups in the mean anchorage loss (*P* > 0.05, Table 3; Fig. 4).

**Table 3:** Posterior mean anchorage loss in the two groups at different time points

Groups	Time 0 Mean ± SD	Time 1 Mean ± SD	Time 2 Mean ± SD	Time 3 Mean ± SD	Time 4 Mean ± SD
Corticotomy	± 1.94 7.4	± 1.88 7.13	± 1.86 6.81	± 1.96 6.51	± 2.04 6.30
Control	± 1.57 7.75	± 1.52 7.15	± 1.50 6.70	± 1.58 6.41	± 1.74 6.14
*P* value	0.93	0.76	0.63	0.65	0.86

One way repeated measures ANOVA

Time 0: Before laser corticotomy; Time 1: 1 month after laser corticotomy, Time 2: 2 months after laser corticotomy, T3: 3 months after laser corticotomy, Time 4: 4 months after laser corticotomy

##### Angle of canine rotation

**Fig. 5:**
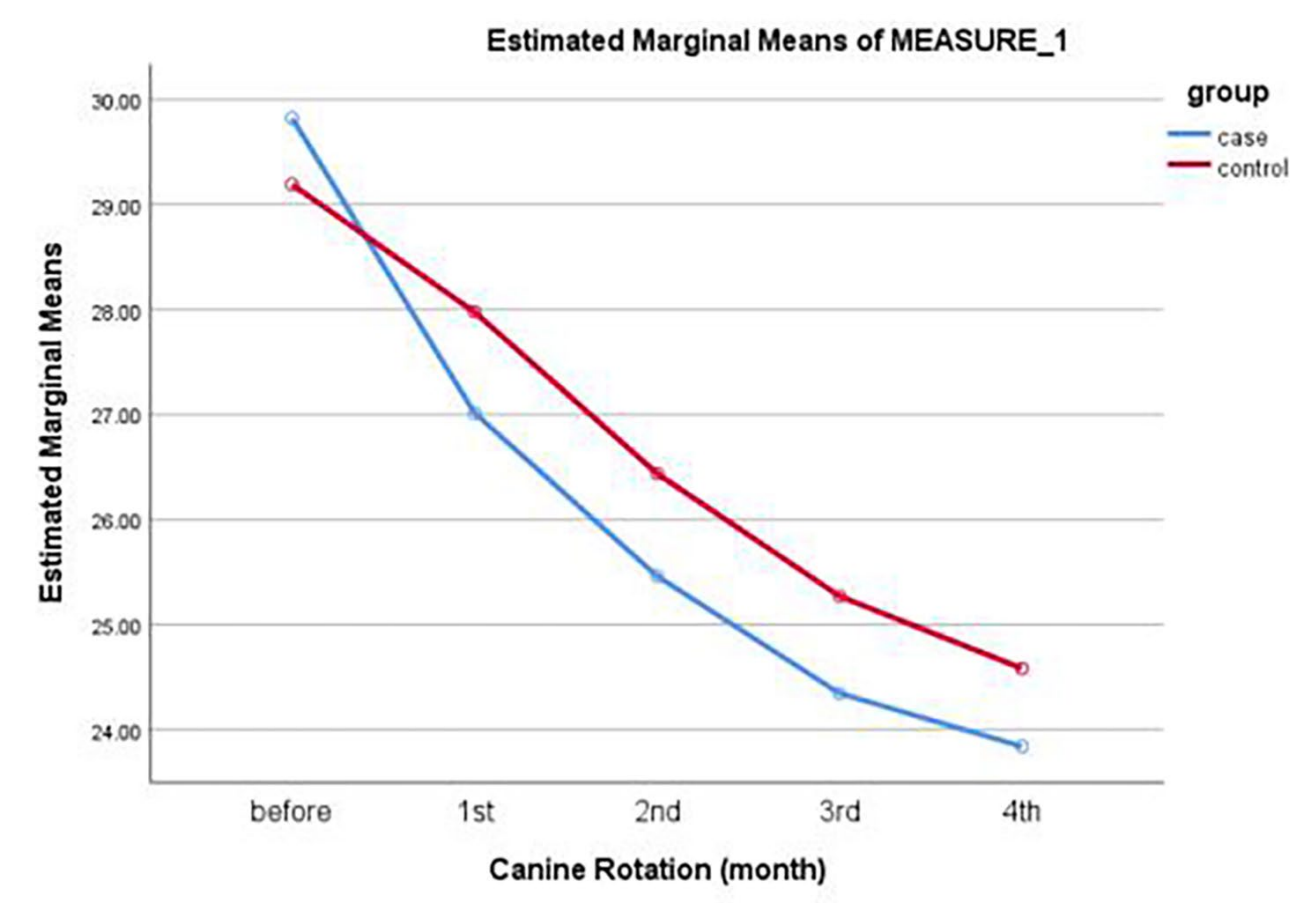
Mean angle of rotation in the two groups at different time points

One-way repeated measures ANOVA showed that the change in angle of canine rotation over time was not significant in any group (*P* = 0.13). Paired t-test showed no significant difference in the mean change in angle of rotation between the two groups (*P* > 0.05, Table 4; Fig. 5).

**Table 4:** Mean angle of rotation in the two groups at different time points

Groups	Time 0 Mean ± SD	Time 1 Mean ± SD	Time 2 Mean ± SD	Time 3 Mean ± SD	Time 4 Mean ± SD
Corticotomy	± 2.45 29.82	± 3.16 27.01	± 3.40 25.46	± 3.43 24.34	± 3.62 23.83
Control	± 2.57 29.19	± 2.72 27.97	± 3.18 26.43	± 3.42 25.26	± 3.62 24.58
*P* value	0.95	0.29	0.39	0.39	0.67

Paired t-test

Time 0: Before laser corticotomy ; Time 1: 1 month after laser corticotomy, Time 2: 2 months after laser corticotomy, T3: 3 months after laser corticotomy, Time 4: 4 months after laser corticotomy

##### PPD

Mann-Whitney test showed no significant difference in the mean PPD between the two groups neither at baseline (before intervention) nor at 4 weeks after the intervention (Table 5, *P* > 0.05).

**Table 5:** Mean PPD in the two groups at different time points

Group	Lateral incisor		Canine		Second premolar	
	Time 0 Mean ± SD	Time 1 Mean ± SD	Time 0 Mean ± SD	Time 1 Mean ± SD	Time 0 Mean ± SD	Time 1 Mean ± SD
Corticotomy	± 0.33 1.2 MD:1 IQR:0.5	± 0.48 1.37 MD:1.25 IQR:0.5	± 0.41 1.08 MD:1 IQR:0.38	± 0.43 1.37 MD:1.25 IQR:0.88	± 0.45 1.75 MD:2 IQR:0.5	± 0.43 1.87 MD:2 IQR:0.5
Control	± 0.32 1.16 MD:1 IQR:0.38	± 0.43 1.37 MD:1.25 IQR:0.88	± 0.22 1.12 MD:1 IQR:0.38	± 0.28 1.41 MD:1.5 IQR:0.38	± 0.53 1.83 MD:1.75 IQR:0.5	± 0.49 1.83 MD:2 IQR:0.5
*P* value	0.75	0.93	0.67	0.67	0.93	0.67

Time 0: Before laser corticotomy; Time 1: 1 month after laser corticotomy

Mann-Whitney test

##### Pain score

Chi-square test showed no significant difference between the two groups regarding the VAS pain score at any time point (Table 6, *P* > 0.05).

**Table 6:** Frequency of different pain severities in the two groups at different time points

Time	Pain score	Corticotomy	Control	*P* value
Day 1	No pain	0%(n:0)	%0 (n:0)	*P* = 0.77
	Mild pain	0%(n:0)	8.3%(n:1)	
	Moderate pain	41.7%(n:5)	33.3%(n:4)	
	Severe pain	50%(n:6)	50%(n:6)	
	Very severe pain	8.3%(n:1)	8.3%(n:1)	
	Worst pain imaginable	0%(n:0)	0%(n:0)	
	Total	100%(n:12)	100%(n:12)	
Day 2	No pain	0%(n:0)	0%(0n:)	*P* = 0.40
	Mild pain	0%(n:0)	8.3%(n:1)	
	Moderate pain	50%(n:6)	58.3%(n:7)	
	Severe pain	50%(n:6)	33.3%(n:4)	
	Very severe pain	0%(n:0)	0%(n:0)	
	Worst pain imaginable	0%(n:0)	0%(n:0)	
	Total	100%(n:12)	100%(n:12)	
Day 3	No pain	0%(n:0)	0%(n:0)	*P* = 0.58
	Mild pain	41.7%(n:5)	41.7%(n:5)	
	Moderate pain	50%(n:6)	58.3%(n:7)	
	Severe pain	8.3%(n:1)	0%(n:0)	
	Very severe pain	0%(n:0)	0%(n:0)	
	Worst pain imaginable	0%(n:0)	0%(n:0)	
	Total	100%(n:12)	100%(n:12)	
Day 4	No pain	0%(n:0)	0%(n:0)	*P* = 0.58
	Mild pain	50%(n:6)	58.3%(n:7)	
	Moderate pain	41.7%(n:5)	41.7% (n:5)	
	Severe pain	8.3%(n:1)	0%(n:0)	
	Very severe pain	0%(n:0)	0%(n:0)	
	Worst pain imaginable	0%(n:0)	0%(n:0)	
	Total	100%(n:12)	100%(n:12)	
Day 5	No pain	%33.3 (n:4)	33.3% (n:4)	*P* = 0.51
	Mild pain	41.7% (n:5)	58.3% (n:7)	
	Moderate pain	25% (n:3)	8.3% (n:1)	
	Severe pain	0% (n:0)	0% (n:0)	
	Very severe pain	0% (n:0)	0% (n:0)	
	Worst pain imaginable	0% (n:0)	0% (n:0)	
	Total	100% (n:12)	100% (n:12)	
Day 6	No pain	58.3% (n:7)	58.3% (n:7)	*P* = 0.57
	Mild pain	33.3% (n:4)	41.7% (n:5)	
	Moderate pain	8.3% (n:1)	0% (n:0)	
	Severe pain	0% (n:0)	0% (n:0)	
	Very severe pain	0% (n:0)	0% (n:0)	
	Worst pain imaginable	0% (n:0)	0% (n:0)	
	Total	100% (n:12)	100% (n:12)	
Day 7	No pain	75% (n:9)	83.3% (n:10)	*P* = 0.59
	Mild pain	16.7% (n:2)	16.7%(n:2)	
	Moderate pain	8.3% (n:1)	0% (n:0)	
	Severe pain	0% (n:0)	0% (n:0)	
	Very severe pain	0% (n:0)	0% (n:0)	
	Worst pain imaginable	0% (n:0)	0% (n:0)	
	Total	100% (n:12)	100% (n:12)	

Chi-square test

##### Pulp vitality

Chi-square test showed no significant difference in pulp vitality of the examined teeth between the two groups neither at baseline (before intervention) nor at 4 weeks after the intervention (Table 7).

**Table 7:** Frequency distribution of pulp vitality test results in the two groups

Response	Lateral incisor				Canine				Second premolar			
	Corticotomy		Control		Corticotomy		Control		Corticotomy		Control	
	Time 0	Time 1	Time 0	Time 1	Time 0	Time 1	Time 0	Time 1	Time 0	Time 1	Time 0	Time 1
Positive	%83.33 (n:10)	%83.33 (n:10)	%100 (n:0)	%100 (n:0)	%91.66 (n:11)	%91.66 (n:11)	%75 (n:9)	%75 (n:9)	%83.33 (n:10)	%83.33 (n:10)	%75 (n:9)	%66.66 (n:8)
Negative	%16.66 (n:2)	%16.66 (n:2)	%0 (n:0)	%0 (n:0)	%8.34 (n:1)	%8.34 (n:1)	%25 (n:3)	%25 (n:3)	%16.66 (n:2)	%16.66 (n:2)	%25 (n:3)	%33.34 (n:4)
Total	100% (n:12)	100% (n:12)	100% (n:12)	100% (n:12)	100% (n:12)	100% (n:12)	100% (n:12)	100% (n:12)	100% (n:12)	100% (n:12)	100% (n:12)	100% (n:12)

Time 0: Before laser corticotomy; Time 1: 1 month after laser corticotomy

Chi-square test

## Discussion

This study assessed the effect of corticotomy with Er: YAG laser on the magnitude of canine retraction. Er: YAG laser was used in the present study since it is minimally invasive, does not require flap elevation, does not cause post-surgical edema, and causes fast gingival healing without scarring. Also, it does not traumatize the interdental papilla and does not cause gingival recession [20].

Previous studies on laser corticotomy are mostly case reports or animal studies, and number of human studies on the efficacy of corticotomy with Er: YAG laser for enhancement of canine retraction is limited [23, 24]. In the present study, anchorage loss, pain, canine rotation angle, and pulp vitality and PPD of canine, lateral incisor, and second premolar teeth were also evaluated. The results showed significantly greater magnitude of canine retraction in the test group than the control group at all-time points. Comparison of speed of retraction showed that in the first 2 months after corticotomy, speed of canine retraction in the test group was significantly greater than that in the control group. The two groups had no significant difference in posterior anchorage loss, indicating that laser corticotomy had no adverse effect on posterior anchorage loss. Canine rotation was slightly greater in the corticotomy group but this difference was not significant with the control group. The differences in pain score, PPD, and vitality tests were not significant between the two groups either.

Alikhani et al. [6], in a clinical trial assessed the effect of micro-osteoperforation on the speed of canine movement and reported significantly greater magnitude of canine retraction in the intervention side than the control side. Their results were in line with the present findings although they used a hand-held appliance for micro-osteoperforation while Er: YAG laser was used for this purpose in the present study. Seifi et al. [20] used Er, Cr: YSGG soft tissue laser for acceleration of OTM in rats and showed significantly greater OTM in the test group than the control group. Their results were in agreement with the present findings despite using a different laser type. Attri et al. [25] evaluated the magnitude of OTM and the perceived pain in the process of acceleration of OTM by micro-osteoperforation. They reported that micro-osteoperforation increased OTM without increasing the pain. Their findings were in accordance with the present results. Abbas et al. [26] evaluated the efficacy of corticotomy with flap and piezocision surgery for acceleration of canine retraction and reported that both methods enhanced OTM. Their results were consistent with the present findings although they made the surgical incisions by using a piezotome. Ali and Salman [21] evaluated the efficacy of Er: YAG laser corticotomy for acceleration of canine movement and reported that the magnitude of canine movement 6 weeks after laser corticotomy was significantly greater than that in the control side, which was similar to the present results. However, they had a short follow-up period of 6 weeks. They also evaluated the pulp vitality of lateral incisors, canines, and second premolars and showed that Er: YAG laser corticotomy had no adverse effect on pulp vitality, which was similar to the present results. Alfawal et al. [27] compared piezocision corticotomy and Er: YAG lasercision for acceleration of canine retraction. The magnitude of canine retraction in both the experimental groups was significantly greater than that in the control group, which was in line with the present findings. They found no significant difference in anchorage loss or canine rotation between the test and control sides, similar to the present findings. Nonetheless, their methodology was different from that of the present study since they made digital photographs from the gypsum casts while the casts were three-dimensionally scanned in the present study. Also, they made five perforations at the extraction site of first premolar. Considering the comparable results of the two studies, it appears that 3 perforations would suffice to obtain favorable results. Mahmoudzadeh et al. [28] used Er, Cr: YSGG laser for acceleration of canine retraction and reported significantly greater canine retraction in the intervention quadrant than the control quadrant, with no significant difference in anchorage loss which was in agreement with the present findings. However, they reported significantly greater canine rotation in the intervention quadrant, which was different from the present findings. This difference may be due to the use of 16 × 22-inch stainless steel wire for canine retraction in their study and subsequently greater play of the wire and bracket, which would result in greater rotation. In the present study, 19 × 25-inch wire was used for canine retraction which decreases the play of the wire and bracket; nonetheless, canine rotation was not significantly increased in the corticotomy side in the present study which indicates that by acceleration of canine retraction through laser corticotomy, canine rotation also increases [28]. Chauhan et al. [29], in their split-mouth clinical trial evaluated the effect of laser corticotomy on OTM. They reported significantly greater canine movement in the laser side than the control side, which was similar to the present findings. In another split-mouth clinical trial, Jaber et al. [30] assessed the effect of Er: YAG laser corticotomy on canine retraction speed within 12 weeks. They reported significantly higher speed of canine retraction during 8 weeks in the laser side than the control side, and the peak retraction in the laser side was at the end of the first month. This value decreased during the second month. Their results were in accordance with the present findings.

In the present study, the magnitude of canine retraction in the first month in the laser side was 2.2 times greater than that in the control side, which was in agreement with the results of Alfawal et al. [27], who also used Er: YAG laser. In their study, the speed of canine retraction in the test side in the first month was almost twice the rate in the control side. Also, in the study by Mahmoudzadeh et al. [28], who used Er, Cr: YSGG laser for this purpose, the rate of canine retraction in the first month in the test group was 2.5 times the rate in the control group, which was in line with the present findings.

In the current study, PPD of lateral incisor, canine, and second premolar teeth was not significantly different between the two groups at any time point, which was in line with the available literature [23, 26, 28].

Pain and discomfort during treatment, especially in the first phase, may affect the patients’ interest in continuation of treatment and the treatment outcome. Several factors can affect pain perception such as age, gender, psychological status and history of patients, previous pain experiences and inter-individual differences in pain perception threshold. Pain is a subjective matter. Thus, the same stimuli can cause different pain intensities in different individuals [31]. It should be also pointed out that the limitation of this study was the impossibility of blinding the participants regarding the side under laser intervention, which may unconsciously affect the patient’s perception of pain. Nonetheless, the present results showed no significant difference in VAS pain scores between the two groups at different time points, which was in agreement with the results of Mahmoudzadeh et al. [28]. Also, laser corticotomy had no adverse effect on pulp vitality of the examined teeth, which was in accordance with the results of Ali and Salman [21].

Split-mouth design was the major strength of the present study which eliminated the confounding effect of inter-individual differences in OTM on the results.

Future studies are required to assess the efficacy of Er: YAG laser corticotomy for OTM in the mandible. Also, future studies are required to compare the efficacy of laser corticotomy with other types of minimally invasive corticotomy to better elucidate the effect of laser on OTM. Furthermore, considering the increase in canine rotation following acceleration of canine retraction by corticotomy, future studies are recommended to assess the speed of canine retraction by applying parallel forces (from the palatal and buccal) to decrease canine rotation.

## Conclusion

Flapless Er: YAG laser corticotomy significantly enhanced canine retraction with no adverse effect on other parameters and no patients’ complication.

### Limitations

In order to obtain the cooperation of patients for future referrals, we were faced with problems such as the need to make frequent calls to patients in order to make necessary arrangements for their attendance at the faculty.

Although no side effects were observed in the period of one month, it is suggested that in future studies, the evaluation of pulp vitality and probing depth be continued for a longer period of time than 4 weeks after corticotomy.

## Data Availability

The datasets generated and analyzed during the current study and are not publicly available due to privacy and ethical concerns but are available from the corresponding author on reasonable request.

## Abbreviations

CONSORT: Consolidated Standards of Reporting Trials
Er:YAG: Erbium-Doped Yttrium Aluminium Garnet
MSP: Medium-short pulse
NiTi: Nickel-Titanium
OTM: Orthodontic Tooth Movement
PPD: Pocket Probing Depth
QSP: Quantum-square pulse
RAP: Regional Acceleratory Phenomenon
VAS: Visual Analog Scale

## References

[1] Segal GR, Schiffman PH, Tuncay OC (2004). Meta analysis of the treatment-related factors of external apical root resorption. Orthod Craniofac Res.

[2] Richter AE, Arruda AO, Peters MC, Sohn W (2011). Incidence of caries lesions among patients treated with comprehensive orthodontics. Am J Orthod Dentofac Orthop.

[3] Zachrisson S, Zachrisson BU (1972). Gingival condition associated with orthodontic treatment. Angle Orthod.

[4] Rosvall MD, Fields HW, Ziuchkovski J, Rosenstiel SF, Johnston WM (2009). Attractiveness, acceptability, and value of orthodontic appliances. Am J Orthod Dentofac Orthop.

[5] Huang H, Williams RC, Kyrkanides S (2014). Accelerated orthodontic tooth movement: molecular mechanisms. Am J Orthod Dentofac Orthop.

[6] Alikhani M, Raptis M, Zoldan B, Sangsuwon C, Lee YB, Alyami B (2013). Effect of micro-osteoperforations on the rate of tooth movement. Am J Orthod Dentofac Orthop.

[7] Frost HM (1983). The regional acceleratory phenomenon: a review. Henry Ford Hosp Med J.

[8] Cruz DR, Kohara EK, Ribeiro MS, Wetter NU (2004). Effects of low-intensity laser therapy on the orthodontic movement velocity of human teeth: a preliminary study. Lasers Surg Med.

[9] Yamaguchi M, Hayashi M, Fujita S, Yoshida T, Utsunomiya T, Yamamoto H (2010). Low-energy laser irradiation facilitates the velocity of tooth movement and the expressions of matrix metalloproteinase-9, cathepsin K, and alpha(v) beta(3) integrin in rats. Eur J Orthod.

[10] Hassan AH, Al-Fraidi AA, Al-Saeed SH (2010). Corticotomy-assisted orthodontic treatment: review. Open Dent J.

[11] Aboul-Ela SM, El-Beialy AR, El-Sayed KM, Selim EM, El-Mangoury NH, Mostafa YA (2011). Miniscrew implant-supported maxillary canine retraction with and without corticotomy-facilitated orthodontics. Am J Orthod Dentofac Orthop.

[12] Liou EJ, Huang CS (1998). Rapid canine retraction through distraction of the periodontal ligament. Am J Orthod Dentofac Orthop.

[13] Sayin S, Bengi AO, Gürton AU, Ortakoğlu K (2004). Rapid canine distalization using distraction of the periodontal ligament: a preliminary clinical validation of the original technique. Angle Orthod.

[14] Işeri H, Kişnişci R, Bzizi N, Tüz H (2005). Rapid canine retraction and orthodontic treatment with dentoalveolar distraction osteogenesis. Am J Orthod Dentofac Orthop.

[15] Alfawal AM, Hajeer MY, Ajaj MA, Hamadah O, Brad B (2016). Effectiveness of minimally invasive surgical procedures in the acceleration of tooth movement: a systematic review and meta-analysis. Prog Orthod.

[16] Showkatbakhsh R, Jamilian A, Showkatbakhsh M (2010). The effect of pulsed electromagnetic fields on the acceleration of tooth movement. World J Orthod.

[17] Kole H (1959). Surgical operations on the alveolar ridge to correct occlusal abnormalities. Oral Surg Oral Med Oral Pathol.

[18] Kim SJ, Moon SU, Kang SG, Park YG (2009). Effects of low-level laser therapy after corticision on tooth movement and paradental remodeling. Lasers Surg Med.

[19] Gantes B, Rathbun E, Anholm M (1990). Effects on the periodontium following corticotomy-facilitated orthodontics. Case reports. J Periodontol.

[20] Seifi M, Younessian F, Ameli N (2012). The innovated laser assisted flapless corticotomy to enhance orthodontic tooth movement. J Lasers Med Sci.

[21] Ali FA, Salman L (2014). Acceleration of canine movement by laser assisted flapless corticotomy [An innovative approach in clinical orthodontics]. J Baghdad Coll Dentistry.

[22] Hoggan BR, Sadowsky C (2001). The use of palatal rugae for the assessment of anteroposterior tooth movements. Am J Orthod Dentofac Orthop.

[23] Wilcko WM, Wilcko T, Bouquot JE, Ferguson DJ (2001). Rapid orthodontics with alveolar reshaping: two case reports of decrowding. Int J Periodontics Restor Dent.

[24] Kravitz ND, Kusnoto B (2008). Soft-tissue lasers in orthodontics: an overview. Am J Orthod Dentofac Orthop.

[25] Attri S, Mittal R, Batra P, Sonar S, Sharma K, Raghavan S (2018). Comparison of rate of tooth movement and pain perception during accelerated tooth movement associated with conventional fixed appliances with micro-osteoperforations - a randomised controlled trial. J Orthod.

[26] Abbas NH, Sabet NE, Hassan IT (2016). Evaluation of corticotomy-facilitated orthodontics and piezocision in rapid canine retraction. Am J Orthod Dentofac Orthop.

[27] Alfawal AMH, Hajeer MY, Ajaj MA, Hamadah O, Brad B (2018). Evaluation of piezocision and laser-assisted flapless corticotomy in the acceleration of canine retraction: a randomized controlled trial. Head Face Med.

[28] Mahmoudzadeh M, Poormoradi B, Alijani S, Farhadian M, Kazemisaleh A (2020). Efficacy of Er,Cr laser incision Corticotomy in Rapid Maxillary Canine Retraction: a Split-Mouth Randomized Clinical Trial. J Lasers Med Sci.

[29] Chauhan D, Datana S, Sharma M, Kumar P, Chopra S (2022). Laser assisted accelerated orthodontics–a split mouth study. Clin Invest Orthod.

[30] Jaber ST, Al-Sabbagh R, Hajeer MY (2022). Evaluation of the efficacy of laser-assisted flapless corticotomy in accelerating canine retraction: a split-mouth randomized controlled clinical trial. Oral Maxillofac Surg.

[31] Briseño-Marroquín B, López-Murillo H, Kuchen R, Casasa-Araujo A, Wolf TG (2021). Pulp sensitivity changes during orthodontic treatment at different time periods: a prospective study. Clin Oral Investig.

